# Abdominal venous thromboses: detection of the *JAK2* p.V617F mutation by next-generation ultradeep sequencing—A prevalence study of patients in Mecklenburg-West Pomerania (2017–2021)

**DOI:** 10.3389/fmed.2023.1344769

**Published:** 2024-01-11

**Authors:** Larissa Henze, Luise Grunwald, Sabine Felser, Maria Witte, Christina Grosse-Thie, Catrin Roolf, Hugo Murua Escobar, Christian Junghanss

**Affiliations:** ^1^Department of Medicine, Clinic III—Hematology, Oncology, and Palliative Medicine, Rostock University Medical Center, Rostock, Germany; ^2^Department of General, Visceral, Thoracic, Vascular, and Transplant Surgery, Rostock University Medical Center, Rostock, Germany

**Keywords:** abdominal venous thromboses, splanchnic vein thrombosis, JAK2 mutation, ultradeep sequencing, anticoagulation

## Abstract

**Background:**

Abdominal venous thromboses are rare thrombotic events with heterogeneous etiologies. They are related to myeloproliferative neoplasms (MPNs) in some patients and can occur as first signs of the disease. MPNs are characterized by mutations in the genes of Janus kinase 2 (JAK2), myeloproliferative leukemia virus oncogene (MPL), and calreticulin (CALR).

**Methods:**

Within the prospective trial “Prevalence of JAK2 mutations in patients with abdominal venous thromboses” (JAK2 MV study; German Clinical Trials Register: DRKS00026943), the peripheral blood of patients with abdominal venous thromboses in Mecklenburg-West Pomerania, a federal state located in north-east Germany, was analyzed by next-generation ultradeep sequencing for MPN-associated mutations. Clinical characteristics and blood cell counts were also of interest. The primary endpoint was the detection of the mutation *JAK2* p.V617F. Secondary endpoints were the detection of other acquired variants of JAK2, as well as MPL and CALR.

**Results:**

A total of 68 patients with abdominal venous thromboses were included from February 2017 to January 2021, with splanchnic veins affected in 65 patients. The mutation *JAK2* p.V617F was present in 13 patients (19%), with four patients showing low variant allele frequencies (VAF 0.1% to 1.9%). The time interval from the thrombotic event to analysis was longer for patients with the mutation. The mutation *MPL* p.W515R was detected in three cases, all of them with low VAF. One patient among them had a concurrent mutation of *JAK2* p.V617F. The mutations *CALR* type I or type II were not found.

**Discussion:**

By analyzing peripheral blood for the mutation *JAK2* p.V617F, an important cause of these rare thrombotic events can be identified. The development of a diagnostic workup with next-generation ultradeep sequencing for the analysis of the *JAK2* p.V617F mutation and further mutations has the potential to better understand the etiology of abdominal venous thromboses in individual patients in regional clinical care, as abdominal venous thromboses are diagnosed by various medical disciplines.

## Introduction

Abdominal venous thromboses are rare events (1). In the literature, hepatic vein thromboses (also known as Budd-Chiari syndrome), portal vein thrombosis, splenic vein thrombosis, and mesenteric vein thromboses are grouped as splanchnic vein thromboses (SVT) (1–4). Incidences range from 0.7 per million people per year for hepatic vein thromboses to 70 per million people for portal vein thromboses (5–7). Thromboses in the abdominal region may also involve the vena cava inferior and other abdominal veins such as renal or ovarian veins. Multiple causes and triggers of abdominal venous thromboses have been described: cirrhosis, inflammatory disease, particularly pancreatitis and inflammatory bowel disease (8), abdominal surgery (9), malignancies (8), myeloproliferative neoplasms (MPN) (1, 10), and congenital or acquired coagulation disorders (1, 11). Abdominal venous thromboses can be complicated by acute sequelae such as liver failure, gastrointestinal bleeding (1), intestinal infarction (12), perforation, peritonitis, and sepsis (11) and can lead to chronic complications, mostly the formation of varices with bleeding risk or re-thrombosis (1, 13). Therefore, in addition to the diagnosis of abdominal venous thrombosis, clarification of causality is fundamental for further treatment decisions and clinical outcomes (2).

The study “Prevalence of JAK2 Mutations in Patients with Abdominal Venous Thrombosis” (JAK2 MV Study) prospectively investigated patients with abdominal venous thromboses in the federal state of Mecklenburg-West Pomerania (MV) in north-east Germany (see Supplementary Figure 1) for the presence of mutations in the gene of Janus kinase 2 (JAK2). The mutation *JAK2* p.V617F represents one of the major diagnostic criteria of MPN (14, 15) and is frequently found in patients with abdominal venous thromboses. JAK2 is a non-receptor tyrosine kinase (16). Activated JAK2 generates the transcription factors known as “Signal transducers and activators of transcription.” This JAK-STAT pathway is involved in the proliferation, differentiation, and self-renewal of the hematopoietic system. The mutation *JAK2* p.V617F constitutively activates the tyrosine kinase (16), leading to increased erythropoiesis and thrombopoiesis and thus resulting in the phenotype MPN (17). Other known driver mutations of MPN, though less commonly detected, exist for myeloproliferative leukemia virus oncogene (*MPL* p.W515R) and calreticulin (*CALR* type I and *CALR* type II) (18). Both of these also result in the activation of the JAK-STAT pathway: mutations in the MPL gene that encodes the thrombopoietin receptor by affecting the intracellular domain of the protein followed by ligand-independent signaling through JAK2, mutations in the CALR gene that alter binding of CALR to MPL associated with JAK2, and thereby over-activating the JAK-STAT signaling pathway (19–22). Both mutations of MPL and CALR were included in the JAK2 MV study as secondary endpoints.

The implementation of the study in the federal state of Germany allows for the depiction of the reality of clinical care for patients with abdominal venous thromboses in a defined region with existing data about population and infrastructure. It also helps to better characterize one of the main causes of abdominal venous thromboses. Potentially, it can lay the groundwork for the development of a diagnostic workup in general clinical care in this area.

## Methods

### Study design

The investigator-initiated JAK2 MV trial was a prospective cohort study to analyze patients in MV with abdominal venous thromboses for the presence of acquired single nucleotide variants in the JAK2 gene. The primary endpoint was the detection of the *JAK2* p.V617F mutation. Secondary endpoints were the detection of other acquired variants of JAK2, as well as the mutations *MPL* p.W515R and *CALR* type I and type II, which are also part of the diagnostic criteria of MPN (14, 15).

Patients in MV with thromboses in hepatic veins, portal veins, splenic veins, mesenteric veins, and/or other abdominal veins were included in the study, regardless of the timepoint of the thrombotic event. For this study, all thrombotic events within 1 month before inclusion were classified as recent events, whereas the others were termed past events. Thrombosis had to be confirmed by imaging [ultrasound, computed tomography (CT), and magnetic resonance imaging (MRI)] or surgically for inclusion in the study. Written informed consent by the patient was required. Patients < 18 years were excluded.

A total of 5 ml of peripheral blood (anticoagulant EDTA) was collected for next-generation sequencing (NGS) and a blood smear for cytology. A standardized recording of demographic, clinical, and laboratory parameters using a questionnaire was within the scope of the study.

The organization of the study, data collection, and analysis, as well as laboratory work (NGS and cytology) were performed at Clinic III—Hematology, Oncology, and Palliative Medicine, Rostock University Medical Center.

The Ethics Committee of Rostock University Medical Center has approved the trial (approval date: 22 November 2016, A 2016–0200), and the trial is registered in the German Clinical Trials Register (DRKS00026943).

### Study implementation

At the beginning of the study, all departments of internal medicine (*n* = 27) and surgery (*n* = 28) at hospitals in MV with at least 80 beds, all specialists in hematology and oncology (*n* = 17), and ~7 months after the start of the study, all specialists in gastroenterology (*n* = 20) were informed about the study by post. In addition to a general brochure about the trial, a patient information sheet, an informed consent form, and a clinical questionnaire were included in the postal mailing. The trial was also promoted at regional educational and scientific meetings.

Patients were informed and included by their treating physicians, who also collected peripheral blood and answered the clinical questionnaire. Blood samples were sent by standard mail. Only upon receipt of the blood sample, together with a valid informed consent form and clinical questionnaire, the analysis of the blood sample was proceeded within the study. Sample processing and analysis were performed pseudonymously.

In cases where a mutation was identified, the respective physician was informed by phone and by written report.

### Next-generation sequencing

A dedicated targeted sequencing gene panel for NGS was designed and validated for use in routine diagnostics. In addition to the total protein-coding sequences of JAK2, the panel also captures the total protein-coding sequences of MPL and CALR. Nucleic acid segments with known mutation hotspots were covered by at least two amplicons in the panel design. Nucleic acid isolation as well as the performance of NGS are outlined in the study by Grunwald et al. (23). Generally, in routine diagnostics for conventional applications, a sensitivity of NGS of approximately 2% is described (24). Here, validation of the above-described NGS panel detected variant allele frequencies (VAF) of 1% and below as the condition of high amplicon coverage (> 2,000) was fulfilled as ultradeep sequencing.

### Statistical analysis

Descriptive participants' characteristics were calculated. Interval-scaled data were tested for normal distribution using the Shapiro-Wilk test. Mean differences were tested using Fisher's exact test or the Mann–Whitney *U*-test. The significance level was set at *p* ≤ 0.050. SPSS 25.0 (SPSS Inc., Chicago, IL, United States) was used for the analysis.

## Results

### Study participants

**Figure 1:**
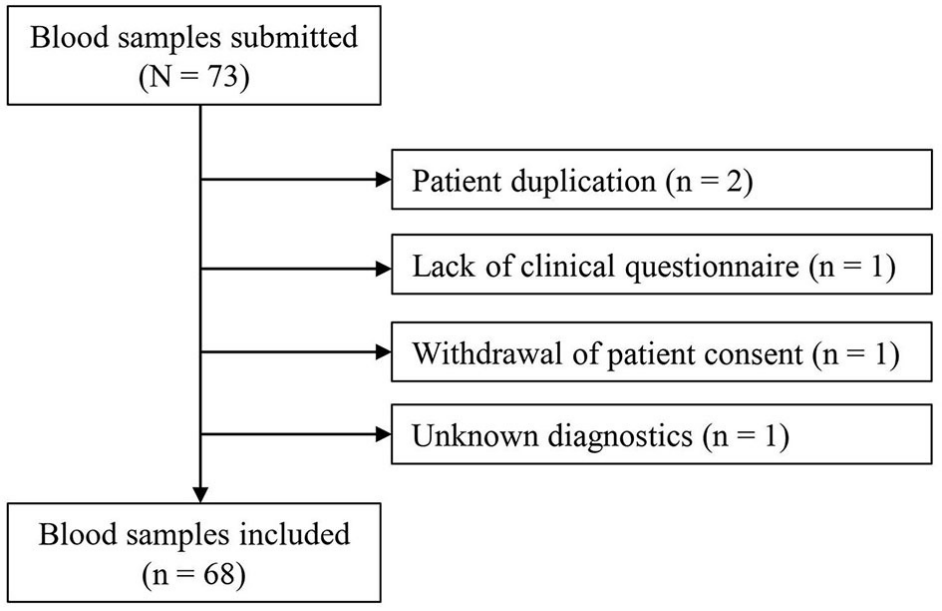
Flowchart of the study. Unknown diagnostics: the diagnostic method for the detection of the thrombosis was not known (Thrombosis had to be confirmed by imaging or surgically for inclusion in the study).

**Figure 2:**
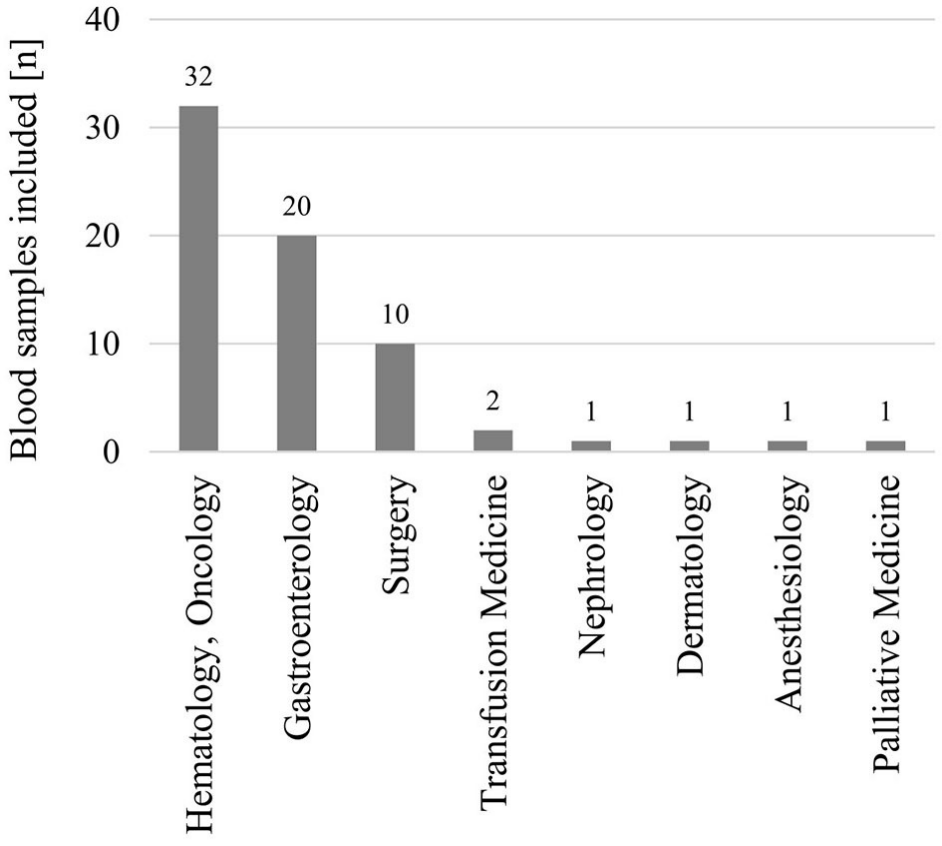
Medical disciplines that submitted blood samples to be included in the study (*n* = 68).

Between 22 February 2017 and 31 January 2021, 73 blood samples were submitted, of which 68 could be included and analyzed in the study (Figure 1). From Rostock originated 84% of samples, and 16% were from different locations in MV (*n* = 4 from Ribnitz-Damgarten, *n* = 2 from Greifswald, *n* = 2 from Stralsund, *n* = 1 from Neubrandenburg, *n* = 1 from Waren, and *n* = 1 from Wismar) (see Supplementary Figure 1). Figure 2 lists the medical disciplines that submitted blood samples.

Patients' characteristics are summarized in Table 1. Overall, 40 (59%) patients were male, 28 (41%) were female. The median age was 62.0 years (range 28–84 years) for male patients and 53.5 years (range 29–80 years) for female patients. In 65 of the 68 patients with abdominal venous thromboses (96%), splanchnic veins were involved. Thrombosis was most commonly localized in the portal vein (*n* = 52), followed by the splenic vein (*n* = 27) and mesenteric veins (*n* = 27). In 33 patients, thromboses were located in two or more abdominal veins. In total, 37 patients had a recent thrombotic event. For the other patients, the thrombotic diagnosis lasted from 32 days to 20 years (in the mean 12 months).

**Table 1:** Characteristics of study participants (*n* = 68).

	**Total**	***JAK2* p.V617F negative**	***JAK2*** **p.V617F positive**		***p* (negative vs. positive)**
	***n** =* **68**	***n** =* **55**	**VAF 0.1–1.9 %*****n** =* **4**	**VAF** ≥**2 %** ***n** =* **9**	
**Sex**, ***n*** **(%)**					0.123
Female	28 (41)	20 (36)	3 (75)	5 (56)	
Male	40 (59)	35 (64)	1 (25)	4 (44)	
**Age (years)**					0.167
Median (min–max)	61 (28–84)	60 (28–84)	69 (60–77)	64 (44–73)	
**Thrombotic site**, ***n***					
Portal vein	52	40	3	9	0.636
Hepatic vein	5	4	0	1	0.22
Splenic vein	27	22	1	3	0.502
Mesenteric vein	27	22	1	3	0.553
SVT	65	53	3	9	0.353
Other veins	9	6	2	1	0.327
**Single vs. multiple sites**, ***n*** **(%)**					0.107
Single site	26 (38)	23 (42)	2 (50)	1 (11)	
Multiple sites	33 (49)	27 (49)	2 (50)	4 (44)	
Unknown	9 (13)	5 (9)	0	4 (44)	
**Date of thrombotic event**, ***n*** **(%)**					**0.041** ^*^
< /= 1 month	37 (54)	33 (60)	2 (50)	2 (22)	
>1 month	29 (43)	20 (36)	2 (50)	7 (78)	
Unknown	2 (3)	2 (4)	0	0	
**Treatment**, ***n***					
Surgery	8	4	1	3	**0.041** ^*^
Thrombolysis°	3	3	0	0	
Anticoagulation	54	45	4	5	0.13
Not specified	7	6	0	1	
**Hepatomegaly**, ***n*** **(%)**					0.142
Yes	10 (15)	9 (16)	0	1 (11)	
No	49 (72)	40 (73)	4 (100)	5 (56)	
Unknown	6 (9)	3 (6)	0	3 (33)	
Not specified	3 (4)	3 (6)	0	0	
**Splenomegaly**, ***n*** **(%)**					0.373
Yes	19 (28)	15 (27)	0	4 (44)	
No	41 (60)	34 (62)	4 (100)	3 (33)	
Unknown	5 (7)	3 (6)	0	2 (22)	
Not specified	3 (4)	3 (6)	0	0	
**Previous thrombosis**, ***n*** **(%)**					0.239
Yes	7 (10)	4 (7)	0	3 (33)	
No	44 (65)	37 (67)	3 (75)	4 (44)	
Unknown	14 (21)	12 (22)	1 (25)	1 (11)	
Not specified	3 (4)	2 (4)	0	1 (11)	
**Thrombophilia**, ***n*** **(%)**					1
Yes	3 (4)	3 (6)	0	0	
No	37 (54)	30 (55)	2 (50)	5 (56)	
Unknown	27 (40)	22 (40)	2 (50)	3 (33)	
Not specified	1 (2)	0	0	1 (11)	
**Malignancy**, ***n*** **(%)**					0.291
Yes°°	21 (31)	19 (35)	1 (25)	1 (11)	
No	34 (50)	25 (46)	2 (50)	7 (78)	
Unknown	12 (18)	10 (18)	1 (25)	1 (11)	
Not specified	1 (2)	1 (2)	0	0	
**Liver cirrhosis**, ***n*** **(%)**					0.193
Yes	8 (12)	5 (9)	0	3 (33)	
No	49 (72)	42 (76)	2 (50)	5 (56)	
Unknown	11 (16)	8 (15)	2 (50)	1 (11)	

SVT, splanchnic vein thrombosis; VAF, variant allele frequency. °No systematic recording of lysis therapy in the JAK2-MV trial. ^°°^ Malignancies reported in patients without detection of the JAK2 p.V617F mutation: (suspected) pancreatic cancer (n = 6), lymphoma, carcinoma of unknown primary (suspected,) gastric cancer (suspected), lung cancer (suspected), cholangiocellular carcinoma, lymphoma (n = 2 each), rectal cancer, intraductal papillary mucinous neoplasia, liver tumor, chronic lymphocytic leukemia (n = 1 each). Malignancies reported in patients with the mutation JAK2 p.V617F are listed in Table 2. ^*^Statistical significant.

Data on the clinical situation were presented by the referring physician and recorded as text on the questionnaire. The presenting symptoms were variable. Pain, gastrointestinal complaints, and liver-associated symptoms were most often mentioned. Of note, the diagnosis was incidental (no symptoms) in 13 patients.

CT was used most frequently (*n* = 54), followed by ultrasound (*n* = 21) and MRI (*n* = 5) as diagnostic procedures. Multiple methodologies were applied to 13 patients. In three patients, diagnosis was established during surgery.

As therapeutic procedure, surgery was performed on eight patients (12%) (Table 1). The use of anticoagulation was reported for 54 (79%) patients in total (Table 1) with the following distribution: low-molecular-weight heparins (LMWH) (*n* = 24), heparin (*n* = 3), phenprocoumon (*n* = 7), direct oral anticoagulants (*n* = 10), and unspecified (*n* = 6). Furthermore, enoxaparin in prophylactic dosage was mentioned for two patients, as was clopidogrel ± acetylsalicylic acid for two patients. For three patients, information about thrombolysis was listed. It has to be noted, though, that this treatment option was not systematically queried in the standardized reporting form (Table 1).

### Prevalence of the mutation *JAK2* p.V617F

The mutation *JAK2* p.V617F was detected in 13 of 68 study participants (VAF 0.2–43%), corresponding to 19%. Four of the thirteen patients had VAF between 0.1% and 1.9%, which is below the conventional cut-off used for routine diagnostics by NGS. The characteristics of study participants with the mutation *JAK2* p.V617F are summarized in Table 2. Strikingly, all but one study participant with the mutation had portal vein thrombosis. Five patients also had thromboses in other abdominal veins, and two patients had experienced a previous thrombotic event. Tumor diseases were reported in these patients as “suspected leukemia” (MPN38; VAF 43.0%), possibly MPN, and carcinoma of the esophagogastric junction (MPN36; VAF 1.4%).

**Table 2:** Patients with the mutation *JAK2* p.V617F: site of thrombosis, previous thrombotic event, malignancy, and liver cirrhosis.

**Code**	***JAK2* p.617F VAF (%)**	**Portal vein**	**Hepatic vein**	**Splenic vein**	**Mesenteric vein**	**Other veins**	**Previous thrombosis**	**Suspected malignancy**	**Liver cirrhosis**
MPN38	43.0	**+**	Unknown	Unknown	Unknown	Unknown	**+**	**+** ^**4**^	**+**
MPN18	31.4	**+**	Unknown	Unknown	Unknown	Unknown	Not specified	-	-
MPN43	28.4 ^6^	**+**	Not specified	Not specified	Not specified	Not specified	**-**	-	**+**
MPN30	24.6	**+**	Unknown	Unknown	Unknown	Unknown	-	Unknown	Unknown
MPN42	17.3	**+**	**+**	**+**	Not specified	-	-	-	-
MPN11	13.2	**+**	Unknown	**+**	**+**	**+** ^**1**^	Unknown	-	-
MPN44	13.2	**+**	-	-	-	-	**+**	-	-
MPN67	12.3	**+**	-	-	**+**	-	**+**	-	**+**
MPN72	4.5	**+**	-	**+**	**+**	-	-	-	-
MPN36	1.4	-	-	-	-	**+** ^**2**^	-	**+** ^**5**^	-
MPN34	0.6	**+**	-	**+**	**+**	Not specified	Unknown	Unknown	Unknown
MPN45	0.3	**+**	-	-	-	**+** ^**3**^	-	-	-
MPN28	0.2	**+**	Not specified	Not specified	Not specified	Not specified	-	-	Unknown

VAF, variant allele frequency; +, yes; –, no. ^1^ venous confluence; ^2^ left renal veins; ^3^ plexus venosus uterinus. ^4^ suspected leukemia; ^5^ carcinoma of the esophagogastric junction. ^6^ in addition *MPL* p.W515R with VAF 0.2%.

**Figure 3:**
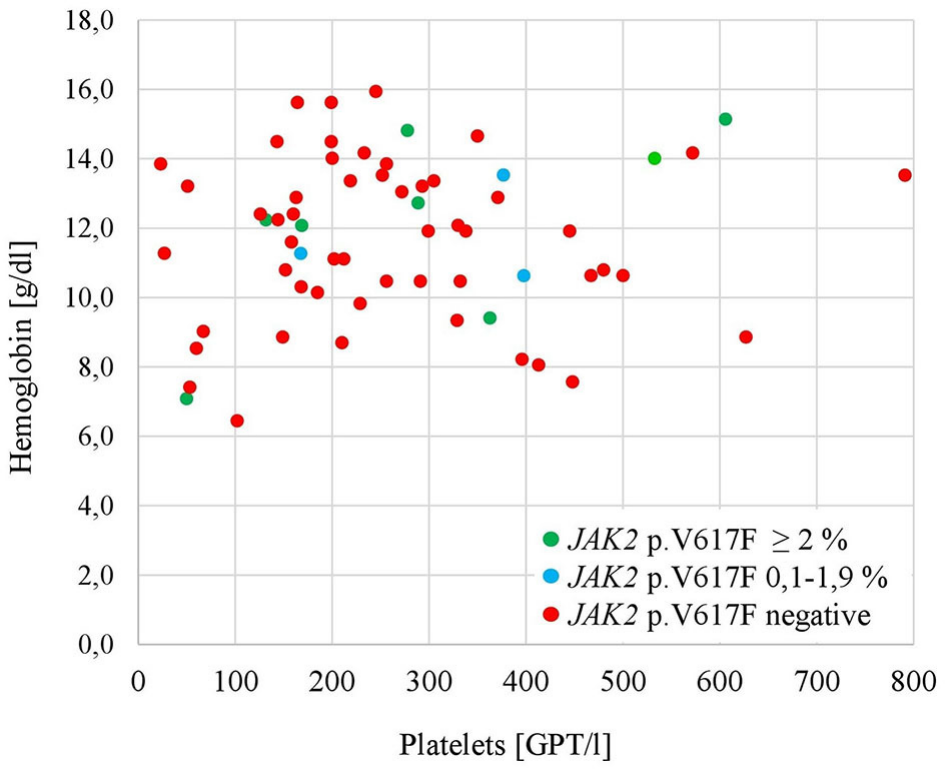
Hemoglobin and platelet count for blood samples included in the study (*n* = 68).

Table 1 presents the characteristics of the total study population for patients without mutation of *JAK2* p.V617F and for patients with *JAK2* p.V617F mutation (VAF 0.1–1.9% and VAF > 2%, respectively). Patients with a mutation of *JAK2* p.V617F, regardless of VAF, were summarized for statistical analysis. Individuals with the mutation were more likely to be female and older. Patients with the mutation also tended more often to have suffered from a previous thrombotic event or to have liver cirrhosis, whereas malignancy was less commonly suspected. Hepatomegaly and splenomegaly were comparable between patients with and without the mutation of *JAK2* p.V617F. Figure 3 visualizes the findings of the hemoglobin and platelet counts of the three groups, showing a scattering of the values without classification of the groups. Patients with the mutation *JAK2* p.V617F had a significantly longer history of abdominal venous thromboses. They were significantly more often treated by surgery (4 of 13 patients). Nine of the thirteen patients with the mutation *JAK2* p.V617F received anticoagulants.

### Prevalence of the mutations *MPL* p.W515R and *CALR* type I and type II

Mutations of *MPL* p.W515R were detected in three cases with VAFs of 1.2%, 0.4%, and 0.2%, thus showing low VAF. The latter patient had a concurrent mutation of *JAK2* p.V617F (MPN43; VAF 28.4%) (Table 2). CALR mutations were not found.

## Discussion

In the literature, in 50%−75% of patients with abdominal venous thromboses, the etiology could be identified (8, 11, 22). In addition to thrombophilia, acquired local and systemic pathophysiological factors are responsible; among the latter, MPNs are of particular importance (3, 22). MPNs associated with abdominal venous thromboses predominantly show the mutation *JAK2* p.V617F (2). In the prospective study “Prevalence of JAK2 mutations in patients with abdominal venous thrombosis” (JAK2 MV study) described here, a prevalence of 19% was found for the mutation *JAK2* p.V617F, whereas the mutation *MPL* p.W515R was only detected in two additional patients (3%) with low VAF. This is in line with other reports about the distribution of the driver mutations in the genes of JAK2, MPL, and CALR in patients with SVT with and without MPN (25–27). While all driver mutations can cause hyperproliferation of myeloid progenitor cells, the mutation *JAK2* p.V617F has been described to contribute to a prothrombotic state by different mechanisms, like enhanced vascular and intercellular adhesion of neutrophils and increased formation of neutrophil extracellular traps (18, 28–30), all of which have not yet been fully elucidated (31). However, as shown in this study and others, the association between the mutation *JAK2* p.V617F and abdominal venous thromboses is clinically evident. Ageno et al. (1) reported a frequency of *JAK2* p.V617F of 20.1 % in a multicentric cohort of patients with SVT with thrombotic events ranging back < 6 months. In the prospective multicenter pilot study for rivaroxaban as a treatment for SVT, *JAK2* p.V617F was detected in 13 of 50 patients tested (26%); patients with Budd-Chiari syndrome or liver cirrhosis were excluded (32). In a monocentric study from 1994 to 2021, published by Colaizzo et al. (13), *JAK2* p.V617F was found in 29.8% by systematic screening of 152 patients at the time of diagnosis of SVT without concomitant cirrhosis or carcinoma. In this study, patients were followed up (median for 64 months, range 1–214 months) for both detection of *JAK2* p.V617F and diagnosis of MPN: 9 of 13 patients with the initial finding of *JAK2* p.V617F and 6 of 8 patients with detection of *JAK2* p.V617F during follow-up were diagnosed with MPN during the course of the study. Analysis of *JAK2* p.V617F was performed using real-time quantitative PCR; the detection limit was defined at 1.5%, but information on VAF was not given.

In the JAK2 MV study, ultradeep sequencing was used to detect and quantify the mutation *JAK2* p.V617F. Given the sensitivity of ultradeep sequencing, VAF from 0.1% to 1.9% could also be described. In the study cohort, four patients showed *JAK2* p.V617F with VAF in this range, and nine patients showed VAF >/= 2% (range 4.5%−43.0%). Patients with evidence of the mutation were diagnosed earlier with abdominal venous thromboses, in line with the observation of Colaizzo et al. (13). How et al. (2, 33) postulate that abdominal venous thromboses are early events, preceding the diagnosis of MPN in some patients. In a Danish population study with nearly 20,000 participants, Cordua et al. (17) showed an increased odds ratio for venous and arterial thromboses in case *JAK2* p.V617F was present with low VAF without a diagnosis of MPN. In light of this, the high sensitivity of the sequencing method is particularly important. In parallel, within the JAK2 MV study, the presence of *JAK2* p.V617F in patients was not associated with hepatomegaly, splenomegaly, or blood count abnormalities (clinical or laboratory signs of MPN). As depicted in Figure 1, hemoglobin levels and platelet counts varied. Particularly hemoglobin levels > 16.5 g/dl or > 16.0 g/dl (referring to men or women), or platelet counts > 450 GPT/l as major diagnostic criteria for MPN (polycythemia vera and essential thrombocythemia, respectively), according to the International Consensus Classification of 2022 (15), were only seen in a few cases with the presence of *JAK2* p.V617F. This implies that high blood cell counts are not the main trigger factors for thrombotic events. The abdominal venous thromboses may influence the development of blood count abnormalities through portal hypertension, inflammation, or bleeding episodes (27). More importantly, as described above, abdominal venous thromboses can be found early before evidence of MPN by diagnostic criteria (14, 15).

Consistent with the literature (2, 13, 33), individuals with abdominal venous thromboses and *JAK2* p.V617F were more likely to be female. Individuals with *JAK2* p.V617F tended to be older. In the literature, comparative data about age refer to individuals with SVT compared to patients with other thromboses (2) or patients with MPN (4, 33). In this regard, patients with SVT tend to be younger.

### Population aspects

The JAK2 MV study had no defined exclusion criteria except for age < 18 years. The study was open to all patients with current or precedent evidence of abdominal venous thromboses in MV. As a consequence, the study cohort was heterogeneous, with competing causes of the thrombotic event, but it represented general clinical care. This highlights the study because, in contrast to other reports, it was not confined to patients of referral centers (1, 7, 12, 13, 34) or MPN or SVT registries (4). Compared to the literature (1, 13), the prevalence of *JAK2* p.V617F in total was lower, but the mutation was still found in about one in five patients. Patients with SVT were represented within the study cohort at 96%.

The study was restricted to MV. The federal state in northeast Germany is an area of low population density with 69 inhabitants per km^2^, consisting of 1.6 million inhabitants (35). In contrast to urban areas, the distribution of medical specialists is limited, and specialized diagnostic centers are scarce. Patients with rare medical problems might be underdiagnosed. Initiating the prospective trial “Prevalence of JAK2 mutations in patients with abdominal venous thromboses” was a step toward distributing highly sophisticated diagnostic tools to potentially all patients in MV.

### Diagnostic aspects

A unique feature of this study was the application of a highly sensitive and quantitative diagnostic method for the detection of the mutation *JAK2* p.V617F: ultradeep sequencing. This allowed the detection of VAF below the sensitivity limitation of NGS or conventional applications such as quantitative polymerase chain reaction (36, 37). Baumeister et al. (37) mention that VAFs of 1% lead to subclinical manifestations of MPN. The JAK2 MV study was not designed to confirm the diagnosis of MPN by further investigation or to follow-up patients. Nevertheless, this aspect is of particular interest for patients with VAF of *JAK2* p.V617F between 0.1% and 1.9%. It should be emphasized that with the detection of *JAK2* p.V617F, further hematological diagnostics regarding MPN are required. This was communicated to the submitting colleagues. Due to the study design, no information about the results was available.

### Limitations

The following limitations of the study have to be acknowledged. First, the study is restricted to MV and might therefore be influenced by the geographic or genetic characteristics of its population. Second, due to the population of MV and the rarity of the thrombotic manifestations, the cohort is small. Still, the results are comparable to data from the literature, taking into account the differences in the inclusion criteria. Third, because the study period ranged from 22 February 2017 to 31 January 2021, the data might be influenced by the COVID-19 pandemic in the last 12 months. During this time, 12 patients were included. Finally, the study was designed as a prevalence study, and a follow-up was not within the scope of the study protocol. Nevertheless, receiving results of further hematological diagnostics would be of great interest for patients with mutations in *JAK2* V617 with VAF > 2% to differentiate MPN from clonal hematopoiesis of indeterminate potential (CHIP) and in those with VAF between 1.0% and 1.9%.

## Conclusion

The JAK2 MV study presents relevant aspects of general clinical care in patients with abdominal venous thromboses: Patients were diagnosed by various medical disciplines. Clinical criteria and blood counts were not conclusive for etiology. By ultradeep next-generation sequencing of peripheral blood samples, the mutation *JAK2* p.V617F was detected in 19% of patients included in the JAK2 MV study. Looking ahead, longitudinal examination of VAF is of interest, as is patients' follow-up with a focus on additional hematological results, especially in the light of evolving data about the pathophysiological influence of CHIP (38). The JAK2 MV study suggests, along with others (38), that “CHIP-associated SVT” (38) or “SVT-predominant MPN” may become a novel clinical entity consisting of patients with abdominal venous thrombosis with the mutation *JAK2* p.V617F. It has therapeutic and prognostic implications before fulfilling the diagnostic criteria of MPN, for example, continuation of anticoagulation, regular surveillance of blood cell counts, and clinical follow-up. Corresponding to the rapid development and distribution of highly sensitive diagnostic procedures like next-generation ultradeep sequencing patients with VAF < 2% will perhaps be included in the future (39). Meanwhile, a larger cohort of patients can contribute to better characterizing different patient groups with abdominal venous thromboses.

### Implications

Patients with abdominal venous thromboses are seen by various medical disciplines.

In a prospective prevalence study in MV, a federal state in Germany, the mutation *JAK2* p.V617F was detected in 19% of patients with abdominal venous thromboses. Clinical or laboratory criteria did not provide evidence for the mutation *JAK2* p.V617F.

Establishing a diagnostic workout for peripheral blood samples in a region can help clarify the etiology of abdominal venous thrombotic events.

Peripheral blood samples are suitable for mutation analysis of *JAK2*. Ultradeep sequencing, as a highly sensitive and quantitative detection method, is advantageous. By using next-generation sequencing, other mutations of interest can be investigated at the same time.

If mutations are detected, further hematological diagnostics should be initiated, and regular follow-up is warranted.

## Data availability statement

The data presented in this study are deposited in the sequence read archive (SRA) by the NIH, accession number SUB10682917.

## Ethics statement

This study involving humans was approved by the Ethikkommission an der Medizinischen Fakultät der Universitaet Rostock, Rostock, Germany. The study was conducted in accordance with the local legislation and institutional requirements. The participants provided their written informed consent to participate in this study.

## Author contributions

LH: Conceptualization, Data curation, Formal analysis, Funding acquisition, Investigation, Project administration, Supervision, Validation, Writing – original draft. LG: Data curation, Formal analysis, Investigation, Methodology, Writing – original draft. SF: Formal analysis, Visualization, Writing – review & editing. MW: Investigation, Writing – review & editing. CGT: Conceptualization, Funding acquisition, Writing – review & editing. CR: Conceptualization, Funding acquisition, Writing – review & editing. HME: Conceptualization, Formal analysis, Funding acquisition, Investigation, Methodology, Supervision, Validation, Writing – original draft. CJ: Resources, Supervision, Writing – review & editing.

## Supplementary material

Supplementary Figure 1 Regional distribution of blood samples included in the study (*n* = 68).
