# Supplementary figures and images for: Abdominal venous thromboses: detection of the JAK2 p.V617F mutation by next-generation ultradeep sequencing—A prevalence study of patients in Mecklenburg-West Pomerania (2017–2021)
Source: Front Med (Lausanne). 2024 Jan 11;10:1344769. doi: 10.3389/fmed.2023.1344769 (PMC10808308; doi:10.3389/fmed.2023.1344769)

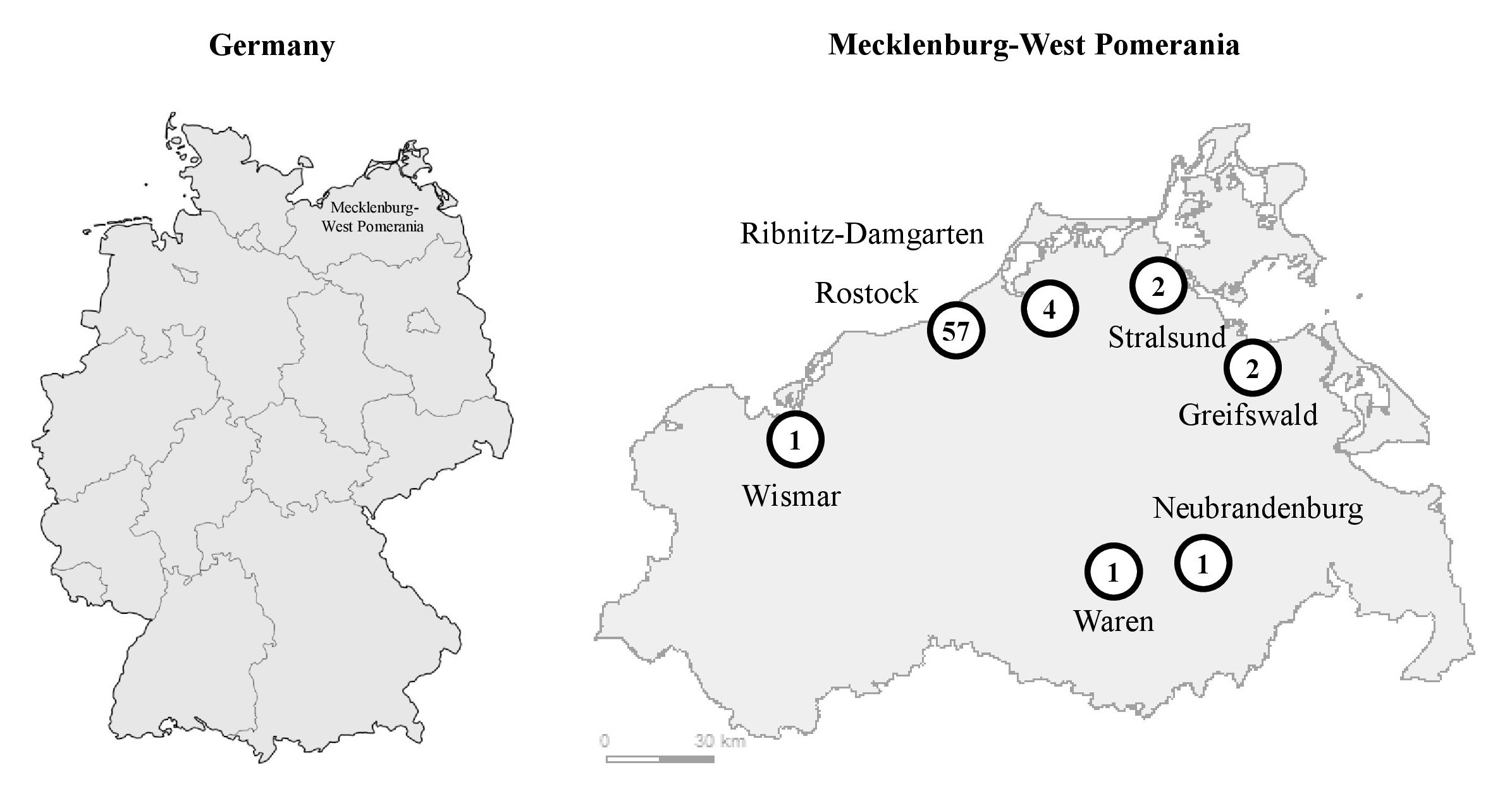

Supplement: Supplementary Figure 1 — Regional distribution of blood samples included in the study (n = 68). [file Image_1.JPEG]
